# Breaking barriers in extensively drug-resistant TB: pretomanid-centred long regimen achieves cure in a bedaquiline-resistant strain

**DOI:** 10.5588/ijtldopen.25.0063

**Published:** 2026-05-11

**Authors:** M. Schiuma, A. Piubello, F. Saluzzo, D. Cattaneo, F. Salari, A. Covizzi, A. Rizzo, F. Fama, C. Ciubotariu, A. Lombardi, S. Antinori, A. Riva, D.M. Cirillo, A. Gori, A. Torre

**Affiliations:** 1 Department of Infectious Diseases, ASST Fatebenefratelli Sacco, Luigi Sacco Hospital, Milan, Italy;; 2 Medical Unit, Damien Foundation, Brussels, Belgium;; 3 Department of Immunology, Transplantation and Infectious Diseases, Vita Salute San Raffaele University, Milan, Italy;; 4 Department of Immunology, Transplantation and Infectious Diseases, IRCCS San Raffaele Scientific Institute, Milan, Italy;; 5 Unit of Clinical Pathology, ASST Fatebenefratelli Sacco University Hospital, Milan, Italy;; 6 Laboratory of Clinical Microbiology, Virology and Bioemergencies, ASST Fatebenefratelli Sacco, Luigi Sacco Hospital, Milan, Italy;; 7 Department of Biomedical and Clinical Sciences “Luigi Sacco”, University of Milan, Milan, Italy;; 8 Centre for Multidisciplinary Research in Health Science (MACH), University of Milan, Milan, Italy.

**Keywords:** tuberculosis, TDM, high-dose bedaquiline, linezolid, XDR-TB

Dear Editor,

Extensively drug-resistant TB (XDR-TB), defined as a strain resistant at least to rifampicin, isoniazid, fluoroquinolones, and bedaquiline or linezolid, remains associated with poor outcomes due to the absence of effective drugs in individualised regimens lasting 18–24 months.^1–3^ A possible explanation for poor outcomes is the intrinsic weakness of the regimen due to the lack of a core drug with bactericidal and sterilising activity (rifampicin, fluoroquinolone, bedaquiline), limited choice of effective companion drugs, and treatment-related toxicity.^4^ Pretomanid, currently approved only within BPaL/BPaLM regimens, has shown strong bactericidal and sterilising activity in preclinical and limited clinical settings, suggesting a potential role beyond standard indications.^3,5^ Linezolid and amikacin have strong early bactericidal and resistance-preventing activity against *Mycobacterium tuberculosis*, but their prolonged use is limited by frequent and sometimes severe toxicity, including myelosuppression, peripheral neuropathy and lactic acidosis with linezolid, and irreversible ototoxicity with amikacin. Therapeutic drug monitoring (TDM) can optimise their dosing, maximising efficacy, while reducing toxicity during extended treatment courses.^6–9^

We report a case of XDR-TB with bedaquiline resistance successfully treated with pretomanid-centred long regimen supported by TDM.

A 51-year-old Italian man, living with HIV since 2010 with lymphocytes CD4^+^ count of 838/mcl at diagnosis, had no history of or contact with TB. In August 2023, after a 7-years loss to follow-up, he presented at his native-city hospital with a 5-months history of fatigue, 20-kg weight loss, dyspnoea, night sweats, and cough. Chest CT revealed bilateral infiltrates and cavitations, and sputum resulted positive for pre-XDR-TB: smear 3+, Xpert® MTB/RIF and MTB/XDR positive with resistance to rifampicin, isoniazid (*katG* and *inhA* mutations), ethionamide (*inhA*), and fluoroquinolones (*gyrA*). Phenotypic tests confirmed resistance to all the first-line drugs. With an HIV viral load of 55,000 copies/mL and lymphocytes CD4^+^ count of 63/mcl, antiretroviral therapy (tenofovir alafenamide/emtricitabine/bictegravir) was reinitiated. A BPaL (bedaquiline/pretomanid/linezolid) regimen was started. Clinical conditions rapidly improved, the sputum smear converted, and the patient was discharged after 45 days. After 1.5 months of correct anti-TB drugs intake, the patient suspended pretomanid and linezolid due to a misunderstanding, but continued with bedaquiline as functional monotherapy for a further 2 months.

When the patient was referred to our HIV-TB outpatient clinic at Sacco Hospital, Milan, a new assessment was done. HIV-RNA was <20 copies/mL and lymphocytes CD4^+^ 687/mcl; genotype (Deeplex® Myc-TB tGS and WGS) and phenotypic testing in MGIT (BD, USA) revealed adjunctive resistance to bedaquiline and clofazimine (*rv0678*, mutation Arg34Leu) – see Table 1. Whole-genome sequencing showed that bedaquiline resistance (*rv0678*, Arg34Leu) was already present in the baseline isolate, belonging to a previously described Northern Italy XDR-TB cluster.^10,11^ An initial regimen including amikacin, meropenem with clavulanic acid, linezolid, delamanid, and terizidone was started in December 2023. After 6 months, the regimen was subsequently modified with pretomanid introduction. As the strain was only tested against bedaquiline critical concentration (1 mg/L), no MIC data were available for bedaquiline. Therefore, high-dose bedaquiline (500 mg for 14 days, followed by 200 mg daily) was introduced for the final 6 months. Subsequent tests determined an MIC of 4 mg/L in MGIT BD (Table 2). Microbiological smear monitoring was performed monthly, also after the end of treatment. Smear and culture on sputum reverted negative after 1 and 2.5 months.

**Table 1.** Baseline microbiological assessment.

Drug	pDST (CC)	Xpert® MTB/RIF-MTB/XDR	Deeplex® Myc-TB and WGS
Rifampicin	R (0.5 mg/L)	R (*rpoB*)	R (Ser450Leu)
Isoniazid	R (0.1 mg/L)	R (*kat*G, *inh*A)	R (Ser315Thr; c-15t)
Pyrazinamide	R (100 mg/L)	NT	R (Ala146Val)
Ethambutol	R (5 mg/L)	NT	R (Met306Ile)
Levofloxacin	R (1 mg/L)	R (*gyr*A)	R (Asp94Tyr)
Moxifloxacin	R (0.25 mg/L)		
Bedaquiline	R (1 mg/L)	NT	R (*rv*0678, Arg34Leu)
Linezolid	S (1 mg/L)	NT	S
Clofazimine	R (1 mg/L)	NT	R (*rv*0678, Arg34Leu)
Pretomanid	S (1 mg/L)	NT	NT
Delamanid	NT	NT	NT
Amikacin	S (1 mg/L)	S	S
Ethionamide	R (5 mg/L)	R (*inh*A)	R (c-15t; insT 4326083)

pDST = phenotypic drug susceptibility testing; CC = critical concentration; WGS = whole-genome sequencing; R = resistant; S = sensitive; NT = not tested.

**Table 2.** Regimens and reasons for modifications.

Therapy		Reason to change the regimen	Microbiology	
Duration	Regimen		Date	Microbiology
1.5 Months	BPaL		Baseline	- Smear positive (3+), culture positive
				- MTB/RIF Ultra: R resistant
				- MTB/XDR: H Fq Eto resistant
				- pDST (only 1st line): RHZE resistant
			34 days	Smear negative, culture positive
55 Days	Bdq	Patient misunderstanding		
3 Months (M1–M3)	Dlm-Lzd-Am-Mpm/Clv-Trd		Baseline	- Smear positive (2+), culture positive
				- MTB/RIF Ultra: R resistant
				- MTB/XDR: H Fq Eto resistant
				- Deeplex: RHZE Fq Bdq Cfz resistant
				- pDST: RHZE Fq Bdq Cfz resistant
			35, 39,46, and 55 days	Smear negative, culture positive
			2.5 months	Smear negative, culture negative
3 Months (M4–M6)	Dlm-Lzd-Am-Trd	Mpm/Clv stopped (culture conversion)	3.5 months	Smear negative, culture negative
6 Months (M7–12)	Pa-Lzd-Trd	Am stopped (sustained culture conversion) – Dlm changed to Pa (higher sterilising activity of Pa)	Monthly	Smear negative, culture negative
6 Months (M13–M18)	Pa-Lzd-Trd-Bdq (high dose)	Bdq high-dose introduction (reinforce sterilising activity)	Monthly	Smear negative, culture negative

BPaL = bedaquiline/pretomanid/linezolid; R = rifampicin; H = isoniazid; Z = pyrazinamide; E = ethambutol; Bdq = bedaquiline; Lzd = linezolid; Fq = fluoroquinolones; Cfz = clofazimine; Dlm = delamanid; Trd = terizidone; Am = amikacin; Mpm/Clv = meropenem/clavulanate; Pa = pretomanid, Eto = ethionamide; pDST = phenotypic drug susceptibility testing; M = month.

Linezolid and amikacin dosing were guided by TDM. For linezolid, 47 trough dosages were performed: 21 (45%) were within the therapeutic range of 0.6–2.0 mg/L (8 [17%] >2 mg/L, 18 [38%] <0.6 mg/L) and the dose administered was modified seven times, ranging from 450 to 1,200 mg (900 mg administered for 10.5 months, 1,200 mg for 4 months, 600 mg for 3 months, 450 mg for 2 weeks), with no onset of side effects related to linezolid, which was continued for the entire 18 months. Amikacin was always administered three times per week (25 mg/kg), 32 peak and 1 trough dosages were performed: 16 (49%) were within the therapeutic range (trough <5 mg/L, peak 65–80 mg/L) and the dose administered was modified five times, ranging from 1,250 to 1,750 mg (1,500 mg being the most frequent administered). Mild ototoxicity with no subjective hearing loss was found by sequential audiometry after around 12 months of treatment, and amikacin was stopped, with subsequent unexpected improvement of ototoxicity. No other adverse events (myelosuppression, kidney and liver failure, QT prolongation, peripheral neuropathy, optic neuritis, or depression) were recorded.

Clinical and radiological improvement occurred, weight increased from 60 to 72 kg and the patient was declared cured after 18 months of treatment. No clinical, radiological, and microbiological evidence of relapse have been detected 6 months after end of treatment.

This case report highlights the difficulties in managing a case with limited treatment options. Fundamental principles have been applied to build a solid TB regimen including a core drug supported by companion drugs with bactericidal and sterilising activity.^4^ By definition, however, an XDR-TB strain is resistant to all currently recognised core drugs (rifampicin, fluoroquinolones, and bedaquiline), leaving available regimens intrinsically weak.^1,4^ Pretomanid is a new nitroimidazole that inhibits cell wall synthesis, only approved as part of BPaL/BPaLM regimen.^1^ Unlike its predecessor, delamanid, preclinical studies have shown that pretomanid has an improved activity against both aerobic and anaerobic mycobacteria,^5^ conferring higher early bactericidal, sterilising, and resistance-preventing activity, although studied only in combination with other agents.^12–14^ These characteristics suggest that pretomanid may be preferable to delamanid in regimens requiring strong sterilising activity^15^ and suggest a possible role as a core drug in a treatment regimen for XDR-TB. Based on this rationale, pretomanid was introduced after detection of bedaquiline resistance, primarily for its sterilising activity and potential role in relapse prevention, as clofazimine was resistant and no other strongly sterilising agents were available. The regimen was completed with linezolid and amikacin with high early bactericidal activity, and meropenem/clavulanate and terizidone.

Given the linezolid and amikacin importance in protecting pretomanid against the development of resistance, and knowing the frequency of side effects during longer regimens, their dosing was guided by TDM. Notably, linezolid dosage of 600 mg was administered for only 3 months, and no side effects have been recorded during the entire regimen duration despite higher dosages administered for most of the treatment. For amikacin, the 25 mg/kg three times/week dosing was preferred over the 15 mg/kg daily dosing to reduce the total week dose (75 vs. 105 mg/kg). Moreover, the dosage was modified to maximise its efficacy. This approach allowed to administer amikacin for almost 12 months before the onset of mild ototoxicity. Finally, high-dose bedaquiline could have had a possible activity during the last 6 months of the treatment: no specific MIC was available for bedaquiline, but only a documented resistance to 1 mg/L concentration. In a possible scenario with an actual MIC of 2–4 mg/L, high-dose bedaquiline could have regained activity. As for now, successful treatment outcome has been reached with no signs of relapse after 6 months of treatment conclusion.

This case suggests that pretomanid may function as a core drug in selected XDR-TB cases when supported by potent bactericidal agents and optimised through TDM. In contexts where bedaquiline and linezolid resistance may be missed, reliance on standardised regimens could accelerate pretomanid resistance. Prospective data are needed, but individualised pretomanid-centred regimens may represent a viable option when no conventional core drug remains.
